# Infant feeding practices and diarrhoea in sub-Saharan African countries with high diarrhoea mortality

**DOI:** 10.1371/journal.pone.0171792

**Published:** 2017-02-13

**Authors:** Felix A. Ogbo, Kingsley Agho, Pascal Ogeleka, Sue Woolfenden, Andrew Page, John Eastwood, Global Child Health Research Interest Group

**Affiliations:** 1 Centre for Health Research, School of Medicine, Western Sydney University, Campbelltown Campus, New South Wales, Australia; 2 Ingham Institute for Applied Medical Research, Liverpool, New South Wales, Australia; 3 School of Science and Health, Western Sydney University, Campbelltown Campus, New South Wales, Australia; 4 Department of Public Health, College of Science, School of Public Health, Health and Engineering, La Trobe University, Bundoora, Victoria, Australia; 5 Department of Community Child Health/Integrated Care, Sydney Children’s Hospital Network, Randwick, New South Wales, Australia; 6 School of Women’s and Children’s Health, The University of New South Wales, Kensington, Sydney, New South Wales, Australia; 7 Menzies Centre for Health Policy, Charles Perkins Centre, School of Public Health, Sydney University, Sydney, New South Wales, Australia; 8 School of Public Health, Griffith University, Gold Coast, Queensland, Australia; 9 Department of Community Paediatrics, Sydney Local Health District, Croydon Community Health Centre, Croydon, New South Wales, Australia; 10 Sydney Children’s Hospital Network, Randwick, New South Wales, Australia; Centre Hospitalier Universitaire Vaudois, FRANCE

## Abstract

**Background:**

The impacts of optimal infant feeding practices on diarrhoea have been documented in some developing countries, but not in countries with high diarrhoea mortality as reported by the World Health Organisation/United Nations Children’s Fund. We aimed to investigate the association between infant feeding practices and diarrhoea in sub-Saharan African countries with high diarrhoea mortality.

**Method:**

The study used the most recent Demographic and Health Survey datasets collected in nine sub-Saharan African countries with high diarrhoea mortality, namely: Burkina Faso (2010, N = 9,733); Demographic Republic of Congo (2013; N = 10,458); Ethiopia (2013, N = 7,251); Kenya (2014, N = 14,034); Mali (2013, N = 6,365); Niger (2013, N = 7,235); Nigeria (2013, N = 18,539); Tanzania (2010, N = 5,013); and Uganda (2010, N = 4,472). Multilevel logistic regression models that adjusted for cluster and sampling weights were used to investigate the association between infant feeding practices and diarrhoea in these nine African countries.

**Results:**

Diarrhoea prevalence was lower among children whose mothers practiced early initiation of breastfeeding, exclusive and predominant breastfeeding. Early initiation of breastfeeding and exclusive breastfeeding were significantly associated with lower risk of diarrhoea (OR = 0.81; 95% confidence interval (CI): 0.77–0.85, P<0.001 and OR = 0.50; 95%CI: 0.43–0.57, respectively). In contrast, introduction of complementary foods (OR = 1.31; 95%CI: 1.14–1.50) and continued breastfeeding at one year (OR = 1.27; 95%CI: 1.05–1.55) were significantly associated with a higher risk of diarrhoea.

**Conclusion:**

Early initiation of breastfeeding and exclusive breastfeeding are protective of diarrhoea in sub-Saharan African countries with high diarrhoea mortality. To reduce diarrhoea mortality and also achieve the health-related sustainable development goals in sub-Saharan African, an integrated, multi-agency strategic partnership within each country is needed to improve optimal infant feeding practices.

## Introduction

In the past three decades, the world has made significant improvements in child survival; however, these gains have been disproportionate between and within countries [1]. Despite significant investments in the prevention of diarrhoeal-related morbidity and mortality, diarrhoea remains one of the leading sources of under-5 mortality (U5M) worldwide, leading to more than 2,100 under-5 deaths daily [2]. Of these deaths, more than three-quarters occur in the mostly poor and less developed countries of the world [3], 42% in sub-Saharan Africa [4]. The impact of diarrhoea-related morbidity and mortality on child survival is most significant in children from developing countries because of suboptimal infant feeding practices, unimproved water and sanitation, a lack of access to vaccination, and inadequate treatment of diarrhoea [3, 5].

In recent years, an increasing number of studies have reported on the benefits of the breast milk and optimal breastfeeding practices for the mother-infant pair [6–9]. These benefits include lower risk for infectious diseases (e.g. diarrhoea) [10, 11], U5M [8], higher intelligence for the infant [11], improved family planning, and reduce risk for breast and ovarian cancers for the mother [6, 12]. Mechanisms for why breast milk is an ‘individualised medicine’ for the infant include stimulation of the infant immune system, maintenance of the microbial changes in the infant’s gastrointestinal system, and stimulation of the epigenetic programming of the infant [13–16]. Given the benefits of optimal breastfeeding to the mother-infant pair, the World Health Organisation and United Nations Children’s Fund (WHO/UNICEF) recommend the initiation of breastfeeding within the first hour of birth and exclusive breastfeeding and introduction of safe and nutritionally adequate complementary foods around the age of six months with continued breastfeeding until 2 years and beyond [17]. Optimal breastfeeding practices are feeding behaviours that are consistent with recommended infant and young child feeding practices (IYCF).

Globally, various attempts have been made to improve IYCF practices, including the International Code of Marketing of Breast milk Substitutes–The Code, the baby friendly hospital initiative–BFHI, and the Global Strategy for Infant and Young Child Feeding. Sub-Saharan Africa has one of the highest prevalence of breastfeeding at one year worldwide; however, only 37% of infants aged less than six months are exclusively breastfed [6]. Lower proportions in infant feeding practices have been reported in many sub-Saharan African countries, where diarrhoea remains a significant source of morbidity and mortality among children under-5 years [18–23]. Plausible reasons attributable to suboptimal exclusive breastfeeding practice in Africa included: lower socio-economic status [20, 23], home birthing [22], culture [20, 24], and poor implementation and monitoring of The Code [20, 25]. Most studies from sub-Saharan Africa that have examined the relationship between infant feeding practices and diarrhoea only focused on early initiation of breastfeeding, exclusive breastfeeding [26], and partial breastfeeding, and these studies were mainly conducted in African countries with lower burden of diarrhoeal mortality [26, 27]. Previously published reports, however, have suggested that other infant feeding behaviours (i.e., predominant breastfeeding, bottle feeding and introduction of solid, semi-solid and soft foods) play significant roles in contributing to the burden of diarrhoeal mortality, particularly in developing countries [28–31].

To date, no studies have investigated the association between these infant feeding practices (i.e. early initiation of breastfeeding, exclusive breastfeeding, predominant breastfeeding, bottle feeding, introduction of solid, semi-solid and soft foods and continued breastfeeding at one year) and diarrhoea in sub-Saharan Africa countries with high diarrhoea mortality using country-level data. It is important to focus on high diarrhoea mortality countries as these are the population with the one of the largest disease burden in the region, and where interventions would have maximum impacts. Thus, this study aimed to investigate the association between infant feeding practices and diarrhoea in sub-Saharan African countries, with high diarrhoea mortality. Evidence based on local data is important to provide timely, culturally-appropriate and context-specific information to advocate for targeted interventions to improve optimal infant feeding practices [31, 32].

In many sub-Saharan African countries, government expenditure as source (i.e., health care spending from the national budget) remains low, [33] despite the commitment made by various national governments to increase funding for the health care sector in the Africa continent. [34] Given this, sub-Saharan Africa receives the largest amount of developmental assistance for health worldwide. Findings from this study will not only be of interest to public health practitioners in sub-Saharan Africa, but also to the international community to assess the scope to which optimal infant feeding practices can influence diarrhoea in sub-Saharan African countries.

## Method

### Data sources

The most recent Demographic and Health Survey (DHS) data for Burkina Faso (2010, N = 9,733); Demographic Republic of Congo (2013; N = 10,458); Ethiopia (2013, 7,251); Kenya (2014, N = 14,034); Mali (2013, N = 6,365); Niger (2013, N = 7,235); Nigeria (2013, N = 18,539); Tanzania (2010, N = 5,013); and Uganda (2010, N = 4,472) were used for this analysis. These countries were selected based on a previous study and a report by the World Health Organisation/United Nations Children Fund which indicated that diarrhoea mortality was highest among these countries in the sub-Saharan African region [3, 35]. The DHS data were collected by country-specific department of health and population in collaboration with Inner City Fund (ICF) International using standardised household questionnaires. A two-stage sampling strategy was employed, where a country was divided into enumeration areas (clusters) based on the census frames in the country, and then, households were randomly selected within each cluster. The DHS datasets were housed within Measure DHS/ ICF International domain and were freely available to apply for online, with all identifier information removed. Permission to use the data was sought from Measure DHS/ ICF International, and approval was granted. Information on household demographics, maternal and child health (including infant and young child feeding practices) were obtained from eligible women aged 15–49 years who were permanent residents in each household surveyed. A total sample of 83,100 maternal responses was used for these analyses, with response rate in the surveys ranging from 96–99%. The DHS provides significant information on infant and young child feeding practices in developing countries [36]. Additional information on the data source and methodology has been described elsewhere in country-specific DHS reports [37].

Diarrhoea was the main outcome in this study, and was defined as the passage of three or more loose or liquid stool per day. Information on childhood diarrhoea was also obtained from mothers, whether each child under-5 years of age in the household had experienced diarrhoea symptoms in the 2 weeks prior to the interview. Additionally, this study restricted analyses to the youngest living child aged less than 24 months, living with respondent (woman aged 15–49 years), and measurement of diarrhoea was based on the child age group for each IYCF practices consistent with a previously published study [31].

The exposure variables were the infants and young feeding indicators (early initiation of breastfeeding, exclusive breastfeeding, predominant breastfeeding, continued breastfeeding at one year and introduction of solid, semi-solid and soft foods), assessed based on the World Health Organisation (WHO) definitions for assessing IYCF practices. [36] For this study, the selection of these indicators was based on previous reports [31, 32, 38].

Early initiation of breastfeeding: The proportion of children 0–23 months of age who were put to the breast within one hour of birth.

Exclusive breastfeeding: The proportion of infants 0–5 months of age who received breast milk as the only source of nourishment, but allowed oral rehydration solution, drops, or syrups of vitamins and medicines.

Predominant breastfeeding: The proportion of infants 0–5 months of age who received breast milk as the main source of nourishment, but allows water, water-based drinks, fruit juice, oral rehydration solution, drops, or syrups of vitamins and medicines.

Continued breastfeeding at one year: The proportion of children 12–15 months of age who were fed breast milk.

Bottle feeding: The proportion of infants 0–23 months of age who received any liquid (including breast milk) or semi-solid food from a bottle with nipple/teat.

Introduction of solid, semi-solid and soft foods: The proportion of infants 6–8 months of age who received solid, semi-solid or soft foods.

A number of potential confounding factors (categorized as socio-economic, health service, individual and household factors) were considered in the analyses based on previously published studies [31, 32]. Socio-economic factors included maternal education, household wealth and maternal employment; and health service factors comprised antenatal care visits and place of delivery. Individual factors included maternal age, child age, sex and gender; and household factors comprised place of residence (i.e. rural or urban), source of drinking water and type of toilet. These socio-economic, health services, individual and household factors varied across countries, and are described in detail in Table 1.

**Table 1:** Characteristics of the study population by country.

	Burkina Faso		Congo DR		Ethiopia		Kenya		Mali		Niger		Nigeria		Tanzania		Uganda	
	N	%	N	%	N	%	N	%	N	%	N	%	N	%	N	%	N	%
***Socio-economic***																		
Mother's employment																		
Not working	1495	26.2	2305	33.9	3097	73.9	1591	47.0	2243	59.0	3957	80.3	4479	38.3	706	22.7	916	31.5
Working	4213	73.8	4491	66.1	1095	26.1	1795	53.0	1559	41.0	973	19.7	7224	61.7	2406	77.3	1993	68.5
Mother's education																		
No schooling	4750	83.2	1234	18.2	2798	66.7	809	11.5	3109	81.8	4203	85.3	5573	47.6	799	25.7	371	12.7
Primary education	621	10.9	2886	42.5	1204	28.7	3845	54.7	335	8.8	474	9.6	2082	17.8	2084	67.0	1863	64.0
Secondary or above	337	5.9	2677	39.4	191	4.5	2370	33.8	358	9.4	248	5.0	4057	34.6	229	7.4	679	23.3
Household wealth																		
Poor	2379	41.7	3004	44.2	1910	45.6	3162	45.0	1539	40.5	1950	39.6	5331	45.5	1407	45.2	1289	44.3
Middle	2465	43.2	2634	38.8	1614	38.5	2482	35.3	1587	41.8	2072	42.0	4337	37.0	1256	40.4	1107	38.0
Rich	866	15.2	1159	17.1	669	16.0	1380	19.7	676	17.8	908	18.4	2044	17.5	450	14.5	517	17.8
***Health service***																		
Frequency of ANC visits																		
None	208	3.6	683	10.1	2373	56.6	276	3.9	954	25.1	617	12.5	4141	35.4	73	2.3	162	5.6
1–3	3629	63.6	2927	43.1	1085	25.9	2908	41.4	1277	33.6	2655	53.9	1556	13.3	1839	59.1	1408	48.3
4 and above	1872	32.8	3185	46.9	735	17.5	3838	54.7	1571	41.3	1658	33.6	6015	51.4	1201	38.6	1343	46.1
Place of birth																		
Home	1484	26.0	1342	19.8	3717	88.6	2425	34.6	1597	42.0	3237	65.7	7296	62.3	1528	49.1	981	33.7
Health facility	4225	74.0	5453	80.3	476	11.4	4591	65.4	2205	58.0	1693	34.4	4416	37.7	1584	50.9	1931	66.3
Postnatal clinic visits																		
None	847	14.8	3536	52.0	3825	91.2	5056	72.0	2067	54.4	2902	58.9	6815	58.2	2098	67.4	1873	64.3
0–2 days	2210	38.7	1027	15.1	143	3.4	1150	16.4	1181	31.1	1328	26.9	3389	28.9	466	15.0	545	18.7
3–42 days	2653	46.5	2234	32.9	225	5.4	818	11.6	554	14.6	701	14.2	1508	12.9	549	17.6	494	17.0
***Individual***																		
Gender																		
Male	2894	50.7	3404	50.1	2166	51.7	3589	51.1	1924	50.6	2478	50.3	5869	50.1	1539	49.5	1448	49.7
Female	2816	49.3	3393	49.9	2027	48.4	3435	48.9	1878	49.4	2452	49.7	5843	49.9	1573	50.6	1465	50.3
Child age (months)																		
0–5	1504	26.4	1935	28.5	1248	29.8	1673	23.8	974	25.6	1480	30.0	2926	25.0	837	26.9	784	26.9
6–11	1469	25.7	1747	25.7	1111	26.5	1871	26.6	1061	27.9	1279	25.9	3198	27.3	793	25.5	811	27.9
18–23	1286	22.5	1753	25.8	1000	23.9	1892	26.9	877	23.1	1331	27.0	3306	28.2	772	24.8	680	23.4
12–17	1451	25.4	1363	20.1	834	19.9	1589	22.6	890	23.4	841	17.1	2282	19.5	710	22.8	637	21.9
Mother's age																		
15–24	2005	35.1	2323	34.2	1282	30.6	2697	38.4	1287	33.8	1618	32.8	3629	31.0	1160	37.3	1120	38.5
25–34	2586	45.3	3201	47.1	2077	49.5	3307	47.1	1840	48.4	2385	48.4	5675	48.5	1342	43.1	1281	44.0
35–49	1118	19.6	1273	18.7	834	19.9	1020	14.5	675	17.8	927	18.8	2408	20.6	611	19.6	511	17.6
***Household***																		
Household location																		
Urban	982	17.2	2104	31.0	557	13.3	2456	35.0	768	20.2	677	13.7	4180	35.7	631	20.3	419	14.4
Rural	4728	82.8	4694	69.1	3635	86.7	4568	65.0	3034	79.8	4254	86.3	7533	64.3	2481	79.7	2493	85.6
^**2**^Type of toilet																		
Not improved	4322	75.8	4220	62.1	3837	91.6	3870	55.6	2302	60.7	4045	82.1	5759	49.4	2646	85.4	934	39.0
Improved	1384	24.3	2573	37.9	354	8.5	3096	44.5	1490	39.3	880	17.9	5909	50.6	451	14.6	1463	61.0
^**1**^Source of drinking water																		
Not improved	1497	26.2	4250	62.5	2332	55.6	2702	38.5	1330	35.0	1677	34.0	5098	43.5	2531	81.3	2438	83.7
Improved	4213	73.8	2547	37.5	1861	44.4	4322	61.5	2472	65.0	3253	66.0	6615	56.5	581	18.7	475	16.3

^**1**^**Source of drinking water**: 'Improved' source of drinking water included residences where water was piped into the dwelling/yard or plot; access to a public tap/standpipe; a tube well or borehole; protected well; protected spring; rainwater and/or bottled water. 'Not improved' water comprised access to an unprotected well; unprotected or spring; tanker truck/cart with drum; surface water; sachet water and/or other source.

^**2**^ **Type of toilet**: 'Improved' type of toilet included toilets such as flush or pour flush toilets or piped to the sewer system, septic tank, pit latrine; flush/pour flush to septic tank; flush/pour flush to pit latrine; ventilated improved pit (VIP) latrine; pit latrine with slab and/or composting toilet. 'Not improved' or shared toilets comprised flush/pour flush not piped to sewer, /septic tank, or /pit latrine; pit latrine without slab/open pit; bucket or hanging toilet/hanging latrine and no facility/bush/field.

The analyses also included a measure of sanitation (type of toilet) and source of drinking water as *a priori* effect measure modifiers to assess if the association between IYCF indicators and diarrhoea differ by levels of sanitation type and drinking water source. In the DHS, the source of water and type of sanitation for each household were obtained by asking respondents about the ‘main source of drinking water’ and the ‘type of toilet facility’ that were used by household members. The source of drinking water and type of toilet were categorised as ‘improved’ or ‘unimproved’ in this study based on the taxonomy of the World Health Organisation and United Nation Children’s Fund Joint Monitoring Programme (JMP) for Water and Sanitation [39]. ‘Improved’ sources of water were defined as a piped water into dwelling, piped water to yard/plot, public tap or standpipe, tube-well or borehole, protected dug well, protected spring or rainwater; while ‘unimproved’ water sources compromised unprotected spring, unprotected dug well, cart with small tank/drum, tanker-truck, surface water or bottled water. ‘Improved’ sanitation facility included a flush toilet, piped sewer system, septic tank, flush/pour flush to pit latrine, ventilated improved pit latrine (VIP), pit latrine with slab, composting toilet, and a special case (i.e., flush/pour flush of excreta to a place unknown the respondent place). ‘Unimproved’ sanitation facility was defined as a flush/pour flush to elsewhere (such as street, yard/plot, open sewer or a ditch), pit latrine without slab, bucket, hanging toilet or hanging latrine, shared sanitation, no facilities, bush or field.

### Statistical analysis

Initial analyses involved a series of frequency tabulations to describe frequencies (and corresponding percentages) for each of the confounding factors (i.e., socioeconomic, health service, individual and household factors) considered in the study. For the pooled analyses, the selected DHS data were combined, and a single model was used to estimate prevalence and standard errors (for calculation of 95% confidence intervals) of infant feeding indicators associated with diarrhoea using the “svy” command to adjust for sampling weights and the cluster sampling employed in the DHS.

Multilevel logistic regression models that adjusted for socio-economic, health service, individual and household factors as common causes (confounders) were used to assess the association between infant feeding practices (i.e., early initiation of breastfeeding, exclusive breastfeeding, predominant breastfeeding, continued breastfeeding at one year, introduction soli, semi-solid and soft food) and diarrhoea in the selected sub-Saharan African countries using “xlogit” command to estimate the relative risks. The modifying effect of the type of sanitation and drinking water sources on the association between infants feeding indicators and diarrhoea was also examined to determine the interaction between the type of toilet use and drinking water sources, and a given infant feeding indicator based on previous reports [31, 32]. These models were restricted to the youngest living child aged less than 24 months living with the respondent (woman aged 15–49 years) to minimise recall bias [31, 40]. All statistical analyses were conducted in STATA version 13.0 (Stata Corporation, College Station, TX, USA).

### Ethics

The DHS project obtained the required ethical approvals from the relevant research ethics committee in each country before the surveys were conducted. The ethical institutions included: Burkina Faso (Burkina Faso National Ethical Committee); Demographic Republic of Congo (Ethics Committee of the Demographic Republic of Congo Ministry of Planning); Ethiopia (National Ethics Review Committee of the Ethiopia Science and Technology Commission); Kenya (Scientific and Ethical review committee of the Kenya Medical and Research Institute); Mali (Ethical Committee of the Faculty of Medicine, Pharmacy and Odonto-stomatology, University of Bamako); Niger (National Consultative Ethics Committee of the Niger Ministry of Health); Nigeria (National Health Research Ethic Committee); Tanzania (National Health Research Ethical Committee); and Uganda (Research and Ethics Committee, Uganda National Council for Science and Technology in Uganda). Participants were informed of the rationale for the surveys, confidentiality of their responses, and that respondents did not need to answer the questions if they do not feel comfortable doing so. Written informed consents were obtained from participants before being allowed in the surveys; and data used in this study were anonymous and are available to apply for online. Measure DHS/ ICF International approved the usage of the data for this study.

## Results

From the analyses, children aged 0–23 months whose mothers engaged in early initiation of breastfeeding and exclusive breastfeeding practices had a lower prevalence of diarrhoea compared to children whose mothers did not engage in early initiation of breastfeeding and exclusive breastfeeding practices, respectively (Table 2). A similar finding was observed in children whose mothers engaged in predominant breastfeeding behaviour. In contrast, children who continued breastfeeding at the age of one year had a high prevalence of diarrhoea compared to children who had stopped breastfeeding. Among infants aged 6–8 months who were introduced to solid, semi-solid and soft foods, a higher proportion experienced diarrhoea compared to their counterparts.

**Table 2:** Prevalence of infant and young child feeding (IYCF) indicators and diarrhoea among children aged 0–23 months in sub-Saharan African countries (N = 83,100).

		Prevalence of IYCF indicators in sub-Saharan African countries	Prevalence of diarrhoea in sub-Saharan African countries
	N*	% (95%LCI-UCI) (a)	% (95%LCI-UCI) (b)
**Early initiation of breast feeding**			
Yes	22,171	44.2 (43.3–45.1)	19.0 (18.2–19.8)
No	28,023	55.8 (54.9–56.7)	20.7 (20.0–21.5)
**Exclusive breast feeding**			
Yes	4,577	29.2 (33.0–35.6)	7.7 (6.6–8.9)
No	11,127	70.8 (69.7–72.0)	13.3 (12.4–14.1)
**Predominant breast feeding**			
Yes	4,891	31.1 (30.0–32.3)	9.6 (8.6–10.73)
No	10,814	68.9 (67.7–70.0)	12.6(11.7,13.5)
**Continued breast feeding at one year**			
Yes	7,583	83.3 (82.2–84.4)	25.4(24.1–26.8)
No	1,520	16.7 (15.6–17.8)	23.2 (20.5–26.1)
**Bottle feeding**			
Yes	4,268	8.5 (8.1–9.0)	21.7 (20.0–23.5)
No	45,926	91.5 (91.0–91.9)	19.78 (19.2–20.4)
**Introduction of solid, semi-solid and softs**			
Yes	4,025	57.6 (55.9–59.2)	25.1 (23.4–27.0)
No	2,970	42.5 (40.8–44.1)	19.5 (17.7–21.4)

N* = weighted total number of children within each IYCF indicators.

(a) Prevalence represents the overall weighted proportion of children for each level (‘Yes’, ‘No’) of infant and young child feeding indicators. (b) Prevalence represents the overall weighted proportion of children with diarrhoea for each level (‘Yes’, ‘No’) of infant and young child feeding indicators.

**Early initiation of breast feeding:** Children 0–23 months of age who were put to the breast within one hour of birth.

**Exclusive Breast feeding:** Infants 0–5 months of age who received breast milk as the only source of nourishment (but allows oral rehydration solution, drops or syrups of vitamins and medicines).

**Predominant breast feeding:** Infants 0–5 months of age who received breast milk as the predominant source of nourishment (but which allows water and water-based drinks fruit juice, ritual fluids, oral rehydration solution, syrups or drops of vitamins).

**Continued breast feeding at one year:** Children 12–15 months of age who were fed breast milk–this indicator includes breast feeding by a wet nurse and feeding expressed breast milk.

**Bottle feeding:** Infants 0–23 months of age who received any liquid (including breast milk) or semi-solid food from a bottle with nipple/teat.

**Introduction of solid, semi-solid and softs:** Infants 6–8 months of age who received solid, semi-solid or soft foods.

In the current study, infants who received breast milk within the first hour of birth were significantly less likely to experience diarrhoea in the selected sub-Saharan African countries compared to those who did not receive breast milk within one of life (Table 3). Similarly, infants aged 0–5 months who received breast milk as the only source of nourishment, but also received oral rehydration solution, drops or syrups of vitamins and medicines were significantly less likely to experience diarrhoea compared to their counterparts. Children aged 12–15 months whose mothers continued breastfeeding were significantly more likely to experience diarrhoea compared to those whose mothers had stopped breastfeeding. Additionally, infants aged 6–8 months whose mothers introduced solid, semi-solid and soft foods were significantly more likely to experience diarrhoea compared to their counterparts. A stratified analysis of the association between infants feeding practices and diarrhoea by each country varied in the analysis (S1 Files).

**Table 3:** Association between infant and young child feeding (IYCF) indicators and diarrhoea in sub-Saharan African countries, with high burden of diarrhoea mortality.

	Unadjusted	P	^¥^Adjusted	P
IYCF indicators	OR 95%(LCI-UCI)	value	OR 95%(LCI-UCI)	value
**Early initiation of breast feeding**				
No	1.00		1.00	
Yes	0.83 (0.79–0.87)	<0.001	0.81 (0.77–0.85)	<0.001
**Exclusive breast feeding**				
No	1.00		1.00	
Yes	0.51 (0.45–0.59)	<0.001	0.50 (0.43–0.57)	<0.001
**Predominant breast feeding**				
No	1.00		1.00	
Yes	1.04 (0.91–1.20)	0.548	1.05 (0.92–1.21)	0.476
**Continued breast feeding at one year**				
No	1.00		1.00	
Yes	1.25 (1.05–1.50)	0.013	1.27 (1.05–1.55)	0.012
**Bottle feeding**				
No	1.00		1.00	
Yes	1.06 (0.98–1.15)	0.151	1.05 (0.97–1.15)	0.223
**Introduction of solid, semi-solid and softs**				
No	1.00		1.00	
Yes	1.27 (1.12–1.44)	<0.001	1.31 (1.14–1.50)	<0.001

^**¥**^Models adjusted for socio-economic factors (maternal education, household wealth and maternal employment); health service factors (antenatal care visit and place of birth); individual factors (maternal age, child’s age and gender) and household factors (household location, source of drinking water and type of toilet).

The analyses testing the modifying effect of sanitation (type of toilet) on the association between infant feeding practices and diarrhoea found that the impact (in terms of effect sizes) of early initiation of breastfeeding and exclusive breastfeeding on diarrhoea prevention were stronger in infants whose households had improved sanitation (Table 4). The analysis also showed that children who continued breastfeeding at one year with unimproved sanitation were more likely to experience diarrhoea compared to their counterparts. A comparable stratified analysis testing the modifying effect of drinking water sources on the association between infant feeding practices and diarrhoea found similar results, where for example, the protective effects (in terms of effect size) of early initiation of breastfeeding and exclusive breastfeeding on diarrhoea were stronger in infants with improved water sources compared to those with unimproved drinking water sources.

**Table 4:** Prevalence and modifying effect of sanitation on infant and young child feeding indicators associated with diarrhoea in sub-Saharan African countries, with high burden of diarrhoea mortality.

	Improved Sanitation						Unimproved sanitation		
IYCF indicators	N*	(%)^$^	OR 95%(LCI-UCI)	P value	N*	(%)^¥^	OR 95%(LCI-UCI)	P value	P for Interaction
**Early initiation of breast feeding**									
No	9583	54.5	1.00		18107	56.7	1.00		0.026
Yes	8016	45.6	0.76 (0.69–0.83)	<0.001	13826	43.3	0.84 (0.79–0.89)	<0.001	
**Exclusive breast feeding**									
No	3821	71.8	1.00		7198	70.6	1.00		0.304
Yes	1504	28.2	0.45 (0.35–0.58)	<0.001	2996	29.4	0.52 (0.44–0.63)	<0.001	
**Predominant breast feeding**									
No	3849	72.3	1.00		6807	66.8	1.00		<0.001
Yes	1475	27.7	1.13 (0.88–1.45)	0.344	3387	33.2	1.02 (0.87–1.21)	0.790	
**Continued breast feeding at 1 year**									
No	717	22.1	1.00		781	13.6	1.00		0.157
Yes	2524	77.9	1.15 (0.86–1.53)	0.338	4954	86.4	1.32 (1.02–1.70)	0.034	
**Bottle feeding**									
No	15369	87.3	1.00		29999	93.9	1.00		<0.001
Yes	2230	12.7	1.03 (0.91–1.18)	0.563	1935	96.1	1.08 (0.96–1.21)	0.205	
**Introduction of solid, semi-solid and soft foods**									
No	1071	42.6	1.00		1883	43.0	1.00		<0.001
Yes	1446	57.5	1.41(1.13–1.78)	0.003	2502	57.1	1.24 (1.04–1.47)	0.014	

N* = total number of children aged 0–23 months with improved and unimproved sanitation.

%^**$**^ **=** proportion of children who engaged in each feeding indicators, but also had improved sanitation.

*%*^**¥**^ *= proportion of children who engaged in each feeding indicators*, *but also had unimproved sanitation*.

Models adjusted for socio-economic factors (maternal education, father’s education, household wealth and maternal employment); health service factors (antenatal care visit); individual factors (maternal age, child’s age and gender) and household factors (household location and source of drinking).

## Discussion

This study found that diarrhoea prevalence was lower among children whose mothers practiced early initiation of breastfeeding, exclusive and predominant breastfeeding. Infants who were introduced to solid, semi-solid and soft foods and those who continued breastfeeding at one year had a higher prevalence of diarrhoea compared to their counterparts. Early initiation of breastfeeding and exclusive breastfeeding were protective against diarrhoea in the selected sub-Saharan African countries, while introduction solid, semi-solid and soft foods and continued breastfeeding were risk factors for diarrhoea. In households with improved sanitary conditions, the protective effect of early initiation of breastfeeding and exclusive breastfeeding against diarrhoea were stronger.

Across various levels of development worldwide, the issue of diarrhoea is most common in the poor and less developed countries, indicating that the problem of diarrhoea is a function of many factors. These factors include: socio-economic status, culture, level of health services or capacity of the primary health care [41], a lack of appropriate health information for diarrhoea prevention, environmental conditions, and basic social amenities such as housing and potable drinking water [42, 43]. Shortages of basic social amenities and dysfunctional health services in many communities in sub-Saharan Africa are the main drivers of diarrhoea-related morbidity and mortality. Changes in health service productivity (including appropriate IYCF messages to mothers), housing availability and affordability, and good drinking water sources, galvanised with strong political support, could substantially reduce the burden of diarrhoea in many sub-Saharan African communities. By improving opportunities for appropriate IYCF behaviours, changes in early initiation of breastfeeding, exclusive breastfeeding and introduction of safe and nutritionally adequate complementary foods can also reduce the burden of diarrhoea in sub-Saharan Africa.

The mechanisms of optimal infant feeding practices in reducing diarrhoea are well-documented [6, 13, 16]. Consistent with prior studies [31, 32, 38, 44], this study demonstrated that early initiation of breastfeeding and exclusive breastfeeding were protective against diarrhoea in sub-Saharan African countries with high diarrhoea mortality. Additionally, a lower likelihood of developing diarrhoea was also found among children who were optimally breastfed (that is, exclusively breastfed from the first hour of birth to six months) in improved sanitary environment and those whose households received improved drinking water. These findings have important operational implications for efforts to reduce diarrhoea mortality in sub-Saharan Africa. Changes in diarrhoea mortality at the population level in Africa would require initiatives in the health, environment and social sectors. In the era of the United Nations millennium developments goals (MDGs), significant improvements were made in providing improved drinking water and sanitation to many communities [39]; however, less attention was given to promotion of infant feeding practices [33]. Current interventions such as the sustainable development goals (SDG 3 and 4), advocating for improved nutrition and healthy lives for all [45], and Global Nutrition Target by 2025 (for example, global target 5 –increasing the rate of exclusive breastfeeding in the first 6 months up to at least 50% by the year 2025) [46] are initiatives needed on a large scale to reduce diarrhoea mortality in resource constraint settings; but these initiatives must be streamlined with available in-country resources to maximise positive results.

Despite international commitments (i.e., the International Code of Marketing of Breast milk Substitutes–The Code, the baby friendly hospital initiative–BFHI, and the Global Strategy for Infant and Young Child Feeding) to improve infant feeding practices [17]; improvement in exclusive breastfeeding has been minimal globally (from 33% in 1995 to 37% in 2014) [47]. Poor implementation and monitoring as well as violation of The Code have been reported in many countries [48–51]. Probable reasons for these practices include: a non-legal binding nature of The Code; non-enforcement of The Code under various national laws, except amended [25]; innovative marketing strategies such as the use of internet channels, including social media [51]; and a lack of training for enforcement officers [52]. These measures have engendered the growth of infant formula marketing worldwide, with subsequent impacts on optimal infant feeding practices [53, 54]. Nonetheless, some authors have argued that rapidly increasing birth rate in developing countries is a major driver for the notable use of infant formula worldwide [55]. In countries (such as Brazil, Bangladesh and Philippines) with stricter regulatory policy framework that limit marketing of infant formula, minimal sales in infant formula have been recorded, indicating that effective regulatory measures can prevent infant formula marketing and improve infant feeding practices in developing countries [56]. Attempts to improve infant feeding practices in sub-Saharan African countries must consider the benefits of effective implementation and monitoring of The Code in the local context.

Existing evidence-based initiatives and policy responses have proven effective in improving infant feeding practices in many developing contexts [57, 58]. Facility-based interventions (such as the BFHI) have also proven to be successful in improving infant feeding practices in many communities, with subsequent impact on diarrhoea mortality [59, 60]. These initiatives, however, have made less impact in many countries in sub-Saharan Africa region compared to developed countries for a number of reasons. These include notable home birthing [61]; myths and beliefs held for infant feeding practices; influence of grandmothers on new mothers whose infant feeding experiences are often based on traditional belief systems [24]; low socio-economic status; and a lack of appropriate health promotion messages and support for nursing mothers [20, 23]. Interventions to improve optimal infant feeding practices with subsequent impact on diarrhoea-mortality in sub-Saharan Africa should be context-specific to maximise outcomes. These strategies will also reduce the impacts of sub-optimal infant feeding practices on other adverse health outcomes for the mother-infant pairs [62].

The study found no association between predominant breastfeeding and diarrhoea in the pooled analysis, but the association varied in each country studied. Previous studies have revealed that predominant breastfeeding (i.e., infants who received breast milk as the main source of nourishment, but received water-based foods) was protective against diarrhoea, and was associated with higher intelligence [63], educational achievement [63, 64], and better income in later life [64]. In addition, empirical evidence that substantially supports exclusive breastfeeding over predominant breastfeeding is limited [65]. Some reports have argued that predominant breastfeeding is a risk factor for diarrhoea, particularly in developing countries [32, 66]. The consumption of contaminated water-based foods in addition to breast milk increases the likelihood of developing diarrhoea in communities where access to potable water and clean sanitary environment is often inadequate [67]. Putting the achievements of the MDGs in context, in relation to provision of drinking water and sanitation in Africa [39]; our study suggests that the association between predominant breastfeeding and diarrhoea vary in sub-Saharan countries, suggesting that interventions to promote infant feeding behaviours must be context-specific, and should consider the socio-economic aspects of the population.

The introduction of solid, semi-solid and soft foods (complementary foods) to infants around the age of six months is recommended because breast milk alone is no longer sufficient to meet the nutritional requirements of the infant; and continued breastfeeding until the child is two years and beyond. Our analysis showed that introduction of complementary foods and continued breastfeeding at one year were associated with higher likelihood of the child experiencing diarrhoea, consistent with previous studies [31, 32]. Evidence suggests that the incidence of diarrhoea among infants in developing countries is highest during the weaning period–a time when complementary foods are introduced to infants [68, 69]. The choice of complementary foods is usually based on the household socio-economic status, culture and infant feeding belief systems [70], presence of a key family member (grandmother) [24], and availability and affordability of local complementary foods [71–73]. Each or a combination of these factors is likely to play a significant role in contributing to onset of diarrhoea in sub-Saharan Africa. For example, a study from Nigeria found that storage, poor preparation and handling of complementary foods, and the addition of locally-sourced condiments significantly contributed to the contamination of complementary foods [74], a major source of childhood diarrhoea in developing countries [69]. Training of health professionals and non-health professionals (traditional birth attendants) on evidence-based initiatives for educating mothers and their families on appropriate infant feeding practices (e.g., complementary foods preparation and handling, and storage) is vital to reducing diarrhoea-related morbidity and mortality in sub-Saharan Africa. Such initiatives (including health promotion messages) should be selected based on the accessibility, availability and affordability of local complementary foods, and should also consider the specific socio-economic environment in which mothers raise their children to ensure sustainability.

Potential limitations of the study should be considered when interpreting the findings. We used self-reported outcome measures, a source of measurement bias. The analyses were based on cross-sectional data, which could make the establishment of a causal relationship between the exposures (infant feeding outcomes) and diarrhoea challenging. Seasonal variations has been reported to influence the incidence of diarrhoea in sub-Saharan Africa [75], and may affect the observed findings given that the NDHS data (employed in this study) were collected at different time points, geographical areas and climatic conditions. Information on the duration and severity of diarrhoea was unavailable in the NDHS dataset. This data would have provided additional information on the level of protection derived from each infant feeding practice.

The study has a number of strengths. The analyses were based on in-country nationally representative samples to ensure adequate generalisability of the study findings, and selection bias is unlikely to influence the observed results given the high response rates (96–99%). The data employed were collected using consistent standardised questionnaires, which provide an important source of information on infant feeding practices and diarrhoea in Africa. [76] This study also provides evidence on the relationship between infant feeding behaviours and diarrhoea in sub-Saharan African countries with the highest diarrhoea mortality in the region to inform context-specific initiatives.

## Conclusion

This study showed that early initiation of breastfeeding and exclusive breastfeeding were protective against diarrhoea, while introduction of solid, semi-solid and soft foods and continued breastfeeding at one year were risk factors for diarrhoea in sub-Saharan African countries with high diarrhoea mortality. There is need for an integrated, multi-agency strategic partnership at all levels within each country to ensure improvement in optimal infant feeding practices, with resultant impact on diarrhoea-related morbidity and mortality as well as achievement of the SDGs.

## Supporting information

S1 Files (DOCX)
