# Supplementary material for: Infant feeding practices and diarrhoea in sub-Saharan African countries with high diarrhoea mortality
Source: PLoS One. 2017 Feb 13;12(2):e0171792. doi: 10.1371/journal.pone.0171792 (PMC5305225; doi:10.1371/journal.pone.0171792)
Supplement: S1 Files — (DOCX) [file pone.0171792.s001.docx]

|  | **Burkina Faso** |  | **Congo DR** |  | **Ethiopia** |  | **Kenya** |  | **Mali** |  |
| --- | --- | --- | --- | --- | --- | --- | --- | --- | --- | --- |
|  | **^¥^**Adjusted |  | **^¥^**Adjusted |  | **^¥^**Adjusted |  | **^¥^**Adjusted |  | **^¥^**Adjusted |  |
| IYCF indicators | OR 95%(LCI-UCI) | P | OR 95%(LCI-UCI) | P | OR 95%(LCI-UCI) | P | OR 95%(LCI-UCI) | P | OR 95%(LCI-UCI) | P |
| **Early initiation of breast feeding** |  |  |  |  |  |  |  |  |  |  |
| No | 1.00 |  | 1.00 |  | 1.00 |  | 1.00 |  | 1.00 |  |
| Yes | 0.94 (0.81-1.10) | 0.473 | 0.89 (0.78-1.01) | 0.078 | 0.81 (0.68-0.95) | 0.012 | 0.89 (0.74-1.06) | 0.194 | 0.79 (0.62-0.99) | 0.040 |
| **Exclusive breast feeding** |  |  |  |  |  |  |  |  |  |  |
| No | 1.00 |  | 1.00 |  | 1.00 |  | 1.00 |  | 1.00 |  |
| Yes | 0.53 (0.33-0.86) | 0.010 | 0.39 (0.270.56) | <0.001 | 0.42 (0.28-0.63) | <0.001 | 0.58 (0.38-0.89) | 0.013 | 0.86 (0.48-1.59) | 0.649 |
| **Predominant breast feeding** |  |  |  |  |  |  |  |  |  |  |
| No | 1.00 |  | 1.00 |  | 1.00 |  | 1.00 |  | 1.00 |  |
| Yes | 1.75 (1.14-2.70) | 0.010 | 1.04 (0.73-1.50) | 0.814 | 1.77 (1.19-2.61) | 0.004 | 0.85 (0.47-1.55) | 0.611 | 0.99 (0.55-1.81) | 0.989 |
| **Continued breast feeding at one year** |  |  |  |  |  |  |  |  |  |  |
| No | 1.00 |  | 1.00 |  | 1.00 |  | 1.00 |  | 1.00 |  |
| Yes | 1.33 (0.38-4.73) | 0.655 | 0.90 (0.54-1.51) | 0.697 | 1.74 (0.66-4.55) | 0.262 | 1.04 (0.40-2.69) | 0.939 | 1.25 (0.31-4.97) | 0.751 |
| **Bottle feeding** |  |  |  |  |  |  |  |  |  |  |
| No | 1.00 |  | 1.00 |  | 1.00 |  | 1.00 |  | 1.00 |  |
| Yes | 1.01 (0.56-1.81) | 0.980 | 1.06 (0.79-1.43) | 0.675 | 0.95 (0.73-1.23) | 0.685 | 0.85 (0.69-1.04) | 0.115 | 1.51 (1.02-2.25) | 0.041 |
| **Introduction of**  **solid, semi-solid and softs** |  |  |  |  |  |  |  |  |  |  |
| No | 1.00 |  | 1.00 |  | 1.00 |  | 1.00 |  | 1.00 |  |
| Yes | 1.16 (0.71-1.89) | 0.557 | 1.98 (1.26-3.10 | 0.003 | 0.97 (0.51-1.83) | 0.925 | 1.01 (0.56-1.82) | 0.963 | 1.31 (0.61-2.78) | 0.480 |

**S1: Association between diarrhoea and infant and young child feeding (IYCF) indicators in sub-Saharan African countries with high burden of diarrhoea mortality**

**^¥^**Models adjusted for socio-economic factors (maternal education, father’s education, household wealth and maternal employment); health service factors (antenatal care visit); individual factors (maternal age, child’s age and gender) and household factors (household location, source of drinking water and type of toilet)

|  | **Niger** |  | **Nigeria** |  | **Tanzania** |  | **Uganda** |  |
| --- | --- | --- | --- | --- | --- | --- | --- | --- |
|  | **^¥^**Adjusted |  | **^¥^**Adjusted |  | **^¥^**Adjusted |  | **^¥^**Adjusted |  |
| IYCF indicators | OR 95%(LCI-UCI) | P | OR 95%(LCI-UCI) | P | OR 95%(LCI-UCI) | P | OR 95%(LCI-UCI) | P |
| **Early initiation of breast feeding** |  |  |  |  |  |  |  |  |
| No | 1.00 |  | 1.00 |  | 1.00 |  | 1.00 |  |
| Yes | 0.83 (0.71-0.98) | 0.028 | 0.57 (0.50-0.66) |  | 1.00 (0.81-1.24) | 0.940 | 0.744 (0.61-0.91) | 0.003 |
| **Exclusive breast feeding** |  |  |  |  |  |  |  |  |
| No | 1.00 |  | 1.00 |  | 1.00 |  | 1.00 |  |
| Yes | 0.70 (0.45-1.09) | 0.111 | 0.58 (0.34-0.99) | 0.048 | 0.40 (0.22-0.73) | 0.003 | 0.38 (0.24-0.60) | <0.001 |
| **Predominant breast feeding** |  |  |  |  |  |  |  |  |
| No | 1.00 |  | 1.00 |  | 1.00 |  | 1.00 |  |
| Yes | 0.79 (0.55-1.12) | 0.187 | 0.76 (0.54-1.06) | 0.110 | 0.45 (0.19-1.06) | 0.0649 | 1.57 (0.73-3.35) | 0.248 |
| **Continued breast feeding at one year** |  |  |  |  |  |  |  |  |
| No | 1.00 |  | 1.00 |  | 1.00 |  | 1.00 |  |
| Yes | 0.87 (0.41-1.81) | 0.702 | 1.41 (0.90-2.23) | 0.129 | 1.89 (0.38-9.34) | 0.437 | 1.80 (0.73-4.42) | 0.205 |
| **Bottle feeding** |  |  |  |  |  |  |  |  |
| No | 1.00 |  | 1.00 |  | 1.00 |  | 1.00 |  |
| Yes | 1.20 (0.77-1.89) | 0.419 | 1.15 (0.97-1.37) | 0.135 | 1.38 (0.84-2.25) | 0.197 | 1.21 (0.96-1.54) | 0.097 |
| **Introduction of**  **solid, semi-solid and softs** |  |  |  |  |  |  |  |  |
| No | 1.00 |  | 1.00 |  | 1.00 |  | 1.00 |  |
| Yes | 0.95 (0.57-1.57) | 0.840 | 1.37 (0.92-2.02) | 0.113 | 1.59 (0.21-1.61) | 0.300 | 3.08 (1.41-6.73) | 0.005 |

**S1: Association between diarrhoea and infant and young child feeding (IYCF) indicators in sub-Saharan African countries with high burden of diarrhoea mortality**

**^¥^**Models adjusted for socio-economic factors (maternal education, father’s education, household wealth and maternal employment); health service factors (antenatal care visit); individual factors (maternal age, child’s age and gender) and household factors (household location, source of drinking water and type of toilet)
